# Phenolic Content, Antioxidant Capacity, and Therapeutic Potential of Mango (*Mangifera indica* L.) Leaves

**DOI:** 10.1002/fsn3.70263

**Published:** 2025-05-10

**Authors:** Mesut Işık, Emrah Dikici, Sevgi Altın, Cemalettin Alp, Kevser Kübra Kırboğa, Ekrem Köksal, Şükrü Beydemir

**Affiliations:** ^1^ Department of Bioengineering, Faculty of Engineering Bilecik Şeyh Edebali University Bilecik Türkiye; ^2^ Science and Technology Application and Research Center Aksaray University Aksaray Türkiye; ^3^ Erzincan Binali Yıldırım University Faculty of Science and Art, Department of Chemistry Erzincan Türkiye; ^4^ Department of Biochemistry, Faculty of Pharmacy Anadolu University Eskişehir Türkiye

**Keywords:** antidiabetic, antioxidant, enzyme inhibition, *Mangifera indica* L, phenolic composition

## Abstract

The phenolic composition, antioxidant capacity, and enzyme inhibition activities of *Mangifera indica* L. leaf (MLs) ethanol extract were comprehensively evaluated to explore its therapeutic and industrial applications. Quantitative profiling of 21 phenolic compounds was performed using the LC–MS/MS method, with vanillic acid (1242.47 μg/L), gallic acid (283.58 μg/L), and quercetin (102.40 μg/L) identified as the most abundant constituents. Antioxidant activities were assessed through DPPH, ABTS, FRAP, and CUPRAC assays, revealing moderate radical scavenging (DPPH: 26.87% ± 2.25%, ABTS: 14.65% ± 1.83%) and metal reduction capacities (FRAP: 0.118 ± 0.07, CUPRAC: 0.172 ± 0.03). In addition, MLs extract demonstrated dose‐dependent inhibitory effects on acetylcholinesterase (AChE, IC_50_: 18.73 μg/mL), butyrylcholinesterase (BChE, IC_50_: 8.56 μg/mL), and α‐glucosidase (IC_50_: 10.83 μg/mL), highlighting its potential in the management of neurodegenerative diseases such as Alzheimer's and metabolic disorders including diabetes. The findings emphasize the bioactive potential of *M. indica* leaves, positioning them as a promising resource for sustainable valorization. By showcasing the applicability of this agricultural by‐product, the study provides a foundation for innovations in the food, nutraceutical, and pharmaceutical sectors. Nevertheless, further in vivo and clinical investigations are essential to fully validate their safety and therapeutic efficacy.

## Graphical abstract

The ethanol extract of *Mangifera indica* L. leaves was evaluated for its phenolic profile (LC–MS/MS), antioxidant capacity (DPPH, ABTS, FRAP, CUPRAC), and enzyme inhibition activities (AChE, BChE, α‐glucosidase). The phenolic compounds exhibiting notable antioxidant activity, the extract shows enzyme inhibitory effects, suggesting it may hold therapeutic potential for conditions such as Alzheimer's disease and diabetes.

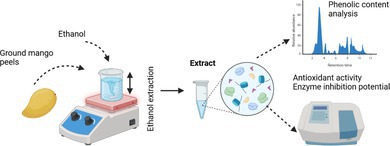

## 1 Introduction

*Mangifera indica* L., a member of the Anacardiaceae family, is recognized as one of the most economically and nutritionally important tropical plants worldwide. Renowned for its vibrant colors, sweet flavor, and rich aroma, mango is also valued for its nutritional composition, which includes high levels of vitamins, minerals, fiber, and bioactive compounds, making it a staple fruit in many regions (Reddy et al. 2021; Tharanathan et al. 2006). Beyond its fruit, the mango plant's leaves, bark, and seeds also hold significant potential, particularly mango leaves (MLs), which are rich in bioactive compounds such as phenolic acids, flavonoids, benzophenone derivatives, and mangiferin. These compounds exhibit notable antioxidant, antimicrobial, and anti‐inflammatory properties, making MLs a promising resource for both health and industrial applications (Berardini et al. 2005; Yahia et al. 2023).

Recent studies have explored the phenolic composition and bioactive properties of MLs, highlighting their health benefits. Principal compounds such as mangiferin (Imran et al. 2017), gallic acid (Kahkeshani et al. 2019), and chlorogenic acid (Mphaphuli Chikhala et al. 2020) have been shown to reduce oxidative stress, regulate lipid metabolism, and mitigate inflammation‐related disorders (Zivković et al. 2024). Additionally, ML extracts have demonstrated potential in managing metabolic diseases through α‐glucosidase inhibition, aiding in diabetes treatment, and acetylcholinesterase (AChE) inhibition, which may benefit neurodegenerative disease management (Infante‐Garcia et al. 2017; Saleem et al. 2019). Similar methodological approaches have been successfully employed in characterizing bioactive compounds from various plant sources. Boga et al. (Boğa et al. 2016) analyzed the phytochemical profiles of *Centaurea* species using liquid chromatography‐electrospray ionization‐tandem mass spectrometry (LC‐ESI‐MS/MS) and evaluated their antioxidant, anticholinesterase, and antimicrobial activities. Their study demonstrated significant radical scavenging activities in methanol extracts and identified quinic acid as the major compound, providing valuable insights into the relationship between phytochemical content and bioactivities. İnci et al. (Inci et al. 2023) investigated Apilarnil, a bee product derived from drone larvae, using comparable techniques including DPPH radical scavenging, metal reducing ability assays, and enzyme inhibition tests against AChE and butyrylcholinesterase (BChE). Their liquid chromatography–tandem mass spectrometry (LC‐MS/MS) analysis identified high concentrations of quinic acid, fumaric acid, and flavonoids such as kaempferol and quercetin, compounds that are also relevant in our investigation of MLs. In a comprehensive study, Ceylan et al. (2021) analyzed 11 *Inula* species, evaluating their phytochemical composition through LC‐MS/MS, antioxidant capacity, and inhibitory effects against key enzymes involved in neurological disorders and diabetes. They found quinic acid to be the predominant compound in most extracts, with a notable presence of other bioactive components such as rutin, chlorogenic acid, and flavonoids. Their multidimensional approach combining chemical profiling with bioactivity assessment provides an excellent framework for understanding the relationship between phytochemical content and the therapeutic potential of medicinal plants. Güven et al. (2023) employed LC‐MS/MS analysis to identify bioactive compounds in *Prunus mahaleb* kernels and evaluated their inhibitory effects on tyrosinase enzyme and melanogenesis, demonstrating a structure–activity relationship among cinnamic acid derivatives. Their study revealed that compounds containing specific structural features exhibited potent non‐competitive tyrosinase inhibition, with potential applications in hyperpigmentation disorders. Altay et al. (2022) investigated *Hypericum scabrum* extracts using LC‐MS/MS analysis and identified 29 phytochemicals, with quinic acid, quercitrin, and isoquercitrin as the major compounds. They evaluated multiple biological activities including antioxidant capacity (DPPH and FRAP), enzyme inhibition (α‐glucosidase and AChE), and anticancer effects against breast cancer cell lines. Their work included molecular docking studies that provided valuable insights into the binding mechanisms of key compounds with target enzymes, strengthening the understanding of structure–activity relationships at the molecular level.

Although the antidiabetic and antineurodegenerative effects of ML extracts under in vivo conditions have been studied in detail in the literature, the relationship between these effects and phenolic compound content has not yet been sufficiently clarified. In this study, the quantitative analysis of phenolic compounds, which form the basis for the biological activities of MLs, was carried out by the LC‐MS/MS method, and the contribution of these compounds to the antidiabetic and antineurodegenerative effects of MLs under in vitro conditions was explored. In this context, the study aims to better understand the potential therapeutic effects of MLs by filling an important gap in the existing literature.

While mango pulp is already well‐known for its nutritional value, studies have increasingly focused on the potential of mango by‐products such as leaves, peel, and seeds in sustainable applications. In this study, ML extract was thoroughly evaluated for its phenolic composition, antioxidant capacity, and enzyme inhibition potential. Using advanced LC‐MS/MS analysis, key phenolic compounds, including gallic acid, fumaric acid, vanillic acid, and quercetin, were identified, and their antioxidant activities were assessed through DPPH, ABTS, FRAP, and CUPRAC assays. The inhibitory effects of ML extract on AChE, BChE, and α‐glucosidase enzymes were also investigated, demonstrating the therapeutic potential of mango leaves in addressing neurodegenerative and metabolic diseases.

## 2 Material and Methods

### 2.1 Chemicals

The reagents utilized in this study included 2,2‐diphenyl‐1‐picrylhydrazyl (DPPH), gallic acid, Folin–Ciocalteu reagent, quercetin, ethylenediaminetetraacetic acid (EDTA), tris(hydroxymethyl)aminomethane (Tris), sodium citrate, and 5,5′‐dithio‐bis (2‐nitrobenzoic acid) (DTNB). These chemicals were procured from Sigma Aldrich, a globally recognized supplier renowned for providing high‐purity and reliable reagents, ensuring the validity and consistency of experimental results. All chemicals were of analytical grade, with purities exceeding 99%, minimizing potential interference and ensuring the accuracy of the assays.

### 2.2 Preparation of the Extract

MLs were collected during the ripening period between September and October 2021 at an altitude of 650 m above sea level in Kozan district of Adana province, Türkiye. The supplied MLs were subjected to air‐drying at ambient temperature in a well‐ventilated setting, ensuring protection from direct sunlight to prevent degradation of bioactive compounds. Following the drying process, the leaves were pulverized into a fine powder using liquid nitrogen to preserve thermolabile constituents and ensure uniform particle size. The powdered plant material was subjected to extraction with ethanol in a 1:10 (w/v) ratio to maximize the yield of ethanol‐soluble phytochemicals. The extraction process was performed on an orbital shaker at room temperature (25°C) for 24 h to ensure adequate diffusion of active compounds into the solvent. After extraction, the mixture was filtered using Whatman No. 1 filter paper to separate the solid residues. The filtrate was then concentrated under reduced pressure using a rotary evaporator to remove the ethanol solvent, producing a crude extract. The resulting extract was dried and stored at 4°C in an airtight container to maintain its stability and prevent contamination (Işık et al. 2015). This prepared extract was subsequently utilized for further biochemical and analytical investigations.

### 2.3 Phenolic Profile of MLs Extract With LC–MS/MS Analysis

The phenolic profile of MLs extract was characterized using liquid chromatography–tandem mass spectrometry (LC‐MS/MS), which was utilized for the quantitative analysis of 21 distinct phenolic compounds. Following the optimization of analytical conditions using high‐purity standard phenolic compounds, the phenolic profiles of plant extracts were comprehensively analyzed. The identification and quantification of phenolic compounds in the plant species were conducted using validated methodologies previously reported in the literature. Specifically, the determination of phenolic compounds in MLs extract was performed according to the method developed by Yilmaz (Kavaz et al. 2021; Yilmaz 2020). Chromatographic separation was achieved using a reverse‐phase C18 Inertsil ODS‐4 column (dimensions: 3.0 mm × 100 mm, particle size: 2 μm). The column temperature was maintained at 40°C, an optimized setting to enhance the resolution of analytes and improve solubility within the mobile phase. The mobile phase was composed of two solvents: water containing 0.1% formic acid (Phase A) and methanol containing 0.1% formic acid (Phase B). The addition of formic acid facilitated ionization of the analytes and mitigated potential corrosion of the ion source. A gradient elution program was employed to optimize the separation of phenolic compounds by adjusting the relative concentrations of the mobile phases. The injection volume for each sample was set at 4 μL, while the flow rate was consistently maintained at 0.5 mL/min to ensure stable chromatographic conditions. Quantitative analyses were performed in triplicate for each compound, and the mean values were calculated to ensure accuracy and reproducibility.

### 2.4 Antioxidant Activity Assays

#### 2.4.1 DPPH Radical Scavenging Activity

The antioxidant potential of the MLs extract was evaluated through its DPPH radical scavenging activity using a slightly modified version of Blois's method, a widely recognized assay for measuring free radical neutralization (Blois 1958). A 0.26 mM solution of 2,2‐diphenyl‐1‐picrylhydrazyl (DPPH) in ethanol was prepared as the working solution. The extract was tested at varying concentrations to assess dose‐dependent activity. Equal volumes of the DPPH solution and extract were mixed and incubated in the dark for 30 min at room temperature to prevent light‐induced degradation of the radical. The activity was monitored by measuring the absorbance at 517 nm using a UV–Vis spectrophotometer. The percentage of DPPH radical scavenging activity was calculated for each concentration, and IC_50_ values were determined by plotting the inhibition percentage against the extract concentration (Al‐Khayri et al. 2022).

#### 2.4.2 ABTS Radical Scavenging Activity

The ABTS radical cation (ABTS^+^) was generated through the oxidation of ABTS (2,2′‐azinobis(3‐ethylbenzothiazoline‐6‐sulfonic acid)) with potassium persulfate, a widely used method for evaluating antioxidant capacity. The reaction mixture was allowed to incubate in the dark for 14 h at room temperature to ensure complete radical formation while avoiding light‐induced decomposition (Re et al. 1999). The resulting ABTS^.+^ solution was then diluted with phosphate buffer or deionized water to achieve an initial absorbance of 0.750 ± 0.025 at 734 nm, a critical step for maintaining consistency across measurements. Various concentrations of the plant extract were mixed with the prepared ABTS^+^ solution, and the decrease in absorbance was recorded at 734 nm using a UV–Vis spectrophotometer. The results were expressed in terms of IC_50_ values, representing the concentration of the extract required to achieve 50% scavenging activity (Kavaz et al. 2022).

#### 2.4.3 Ferric Reducing Power Assay

Ferric Reducing Power Assay was employed to evaluate the electron‐donating capacity of the extract, an indicator of its overall antioxidant potential. This method is based on the reduction of the Fe^3+^/ferricyanide complex to Fe^2+^ in the presence of antioxidants, which subsequently forms a blue‐colored ferrous tripyridyltriazine complex measurable at 700 nm. In this assay, the extract was initially mixed with 0.2 M phosphate buffer (pH 6.6) and 1% potassium ferricyanide [K_3_Fe(CN)_6_], followed by incubation at 50°C for 20 min to facilitate the reduction reaction. After incubation, 10% trichloroacetic acid (TCA) was added to terminate the reaction, and the mixture was centrifuged to separate the precipitate. The resulting supernatant was combined with FeCl_3_ solution to form the ferric‐ferrous complex. The absorbance of the mixed solution was measured spectrophotometrically at 700 nm (Oyaizu 1986; Yüksel et al. 2021).

#### 2.4.4 Cupric Reducing Antioxidant Capacity (CUPRAC)

The cupric reducing antioxidant capacity (CUPRAC) assay was employed to assess the antioxidant activity of the extract, based on its ability to reduce Cu^2+^ ions to Cu^+^ ions. This reduction occurs in the presence of neocuproine, which forms a stable orange‐colored Cu^+^‐neocuproine complex with maximum absorbance at 450 nm. In this assay, the reaction mixture consisted of 1 mL of 10 mM CuCl_2_ solution, 1 mL of 7.5 mM neocuproine solution, and 1 mL of ammonium acetate buffer (1 M, pH 7.0). Different concentrations of the MLs extract were added to the reaction mixture, and the total volume was adjusted to 4 mL with distilled water (Apak et al. 2006; Ndhlala et al. 2024). The mixture was incubated for 30 min at room temperature to ensure complete reaction and was measured spectrophotometrically at 450 nm.

#### 2.4.5 Cholinesterase Inhibition Assay

The inhibitory activity of the extract against AChE and BChE enzymes was assessed using the Ellman method, a widely adopted spectrophotometric assay for cholinesterase inhibition studies (Ellman et al. 1961). The reaction mixture consisted of the enzyme solution, Tris–HCl buffer (pH 8.0), DTNB, and the substrate (acetylthiocholine iodide for AChE or butyrylthiocholine iodide for BChE). The extract was added at varying concentrations to evaluate its dose‐dependent inhibitory effects. The mixture was incubated for a specific duration at 25°C to allow enzymatic hydrolysis to occur. Absorbance readings at 412 nm were recorded using a UV–Vis spectrophotometer to quantify the formation of the yellow‐colored product. IC_50_ values, representing the concentration of the extract required to inhibit 50% of the enzyme activity, were calculated from the dose–response curves (NecİP and Işık 2019; Türkeş et al. 2022). The assay was performed in triplicate for each concentration to ensure accuracy and reproducibility of the results.

#### 2.4.6 α‐Glucosidase Inhibition Assay

The inhibitory potential of the extract on α‐glucosidase enzyme activity was evaluated using p‐nitrophenyl‐D‐glucopyranoside (p‐NPG) as the substrate, a widely used method for assessing α‐glucosidase inhibitors. The reaction mixture included the enzyme solution, the extract at varying concentrations, and the substrate dissolved in an appropriate buffer (e.g., phosphate buffer, pH 6.8). Absorbance of the resulting solution was recorded at 405 nm using a UV–Vis spectrophotometer (Tao et al. 2013; Kaya et al. 2024). The inhibitory activity of the extract was calculated as a percentage of enzyme inhibition relative to a control without the extract. IC_50_ values, representing the concentration of the extract required to inhibit 50% of α‐glucosidase activity, were determined from dose–response inhibition curves (Demir et al. 2017).

### 2.5 Statistical Analysis

Each experiment was performed in triplicate to ensure the reliability and reproducibility of the results. All experiments were performed in triplicate to ensure the reliability and reproducibility of the results. Data are expressed as the mean ± standard deviation (SD). Statistical analyses were conducted using GraphPad Prism version 8 software, which provides robust tools for data visualization and hypothesis testing.

## 3 Results

### 3.1 Phenolic Profile of MLs Extract With LC‐MS/MS Analysis

Table 1 comprehensively presents the phenolic compounds in the ethanol extract of MLs as determined by LC‐MS/MS analysis. A total of 21 phenolic compounds were analyzed, among which vanillic acid (1242.47 μg/L), fumaric acid (378.29 μg/L), gallic acid (283.58 μg/L), butein (283.32 μg/L), and quercetin (102.40 μg/L) were identified as the most abundant. Other compounds detected at lower concentrations included catechin hydrate (5.08 μg/L), resveratrol (8.47 μg/L), and phloridzin dihydrate (7.16 μg/L), while some compounds, such as oleuropein, ellagic acid, and luteolin, were not detected. The limits of detection (LOD) and quantification (LOQ) ranged from 0.05 μg/L to 207 μg/L, demonstrating the high sensitivity of the method. Recovery rates close to 100% further confirmed the efficiency of the extraction process and the reliability of the analysis. Additionally, the determination coefficients (*R* ^2^ values) for all compounds were ≥ 0.996, indicating the accuracy of the calibration curves (Table 1). Validation parameters of phenolic compounds determined by LC‐MS/MS are presented in Table S1 and Figure S1. These findings demonstrate that MLs extract is rich in phenolic compounds, which significantly contribute to its bioactivity.

**TABLE 1:** The Phenolic content of *Mangifera indica* L. ethanol extract.

Standard compounds	LOD/LOQ (μg/L)	Recovery (%)	RT	Concentration (μg/L)
Quercetin	22.5/25.7	1.001	3886	102.40
Acetohydroxamic acid	2.8/8.2	1.000	0396	62.71
Catechin hydrate	8.2/11.4	0.994	2712	5.08
Vanillic acid	125.5/142.2	1.001	3519	1242.47
Resveratrol	9.0/13.6	0.998	3192	8.47
Fumaric acid	25.2/31.3	0.997	0454	378.29
Gallic acid	0.90/1.6	1.000	1.418	283.58
Caffeic acid	6.3/10.7	1.009	3114	3.65
Phloridzin dihydrate	61.0/207.0	1.000	3450	7.16
Oleuropein	0.05/1.0	0.997	3.567	N.D.
Ellagic acid	0.101/0.333	1.002	3900	N.D
Myricetin	55.4/59.6	0.999	5.010	14.92
Protocatechuic acid	30.3/35.4	1.011	3.556	N.D.
Butein	22.7/28.6	0.096	3923	283.32
Naringenin	5.4/6.4	0.998	4483	N.D.
Luteolin	0.5/2.5	1.007	4124	N.D.
Kaempferol	206.6/214.3	0.999	4115	N.D.
Alizarin	65.2/77.5	0.966	4.594	N.D.
4‐Hydroxybenzoic acid	30.5/40.25	0.996	4235	N.D.

Abbreviations: LOD/LOQ (μg/L), limit of detection/limit of quantitation; ND, not detected; RT, retention time.

### 3.2 Radical Scavenging and Metal Reduction Activities of MLs Ethanol Extract

Table 2 presents the radical scavenging and metal reduction activities of MLs ethanol extract, evaluated through DPPH, ABTS, FRAP, and CUPRAC assays. The DPPH radical scavenging activity of the extract was measured as 26.866% ± 2.25%, while the ABTS radical scavenging activity was determined to be 14.653% ± 1.83%. These values were significantly lower compared to the standard antioxidants BHA (DPPH: 73.36% ± 4.12%, ABTS: 83.35% ± 7.08%) and BHT (DPPH: 49.62% ± 4.26%, ABTS: 46.19% ± 2.98%). In the FRAP assay, which evaluates metal reduction capacity, the extract exhibited an absorbance value of 0.118 ± 0.07, which was lower than those of the standards BHA (0.39 ± 0.05) and Trolox (0.25 ± 0.01). Similarly, in the CUPRAC assay, the extract showed an absorbance value of 0.172 ± 0.03, which was also lower compared to BHA (0.58 ± 0.04) and Trolox (0.54 ± 0.03).

**TABLE 2:** Radical removal and metal reduction activity of *Mangifera indica* L.

Antioxidants	DPPH^a^ (0.2 μg mL^−1^)	ABTS^a^ (0.2 μg mL^−1^)	FRAP assay^b^ (0.2 μg mL^−1^)	CUPRAC assay^b^ (0.2 μg mL^−1^)
*Mangifera indica* L.	26.866 ± 2.25	14.653 ± 1.83	0.118 ± 0.07	0.172 ± 0.03
BHA	73.36 ± 4.12	83.35 ± 7.08	0.39 ± 0.05	0.58 ± 0.04
BHT	49.62 ± 4.26	46.19 ± 2.98	0.67 ± 0.07	0.61 ± 0.04
Trolox	78.53 ± 6.97	82.18 ± 6.81	0.25 ± 0.01	0.54 ± 0.03

*Note:* Standard antioxidants (BHA, butylated hydroxyanisole; BHT, butylated hydroxytoluene, trolox).

^a^ Values are expressed as percent radical scavenging activity.

^b^ Values are expressed as absorbance. High absorbance indicates high metal reduction capacity.

These results indicate that the radical scavenging and metal reduction activities of MLs extract are limited and significantly lower than those of standard antioxidants. However, the extract still demonstrates a certain level of antioxidant capacity, suggesting the presence of bioactive compounds that contribute to its natural antioxidant properties.

### 3.3 Inhibition Activities of MLs Extract Against AChE, BChE, and α‐Glucosidase

**FIGURE 1:**
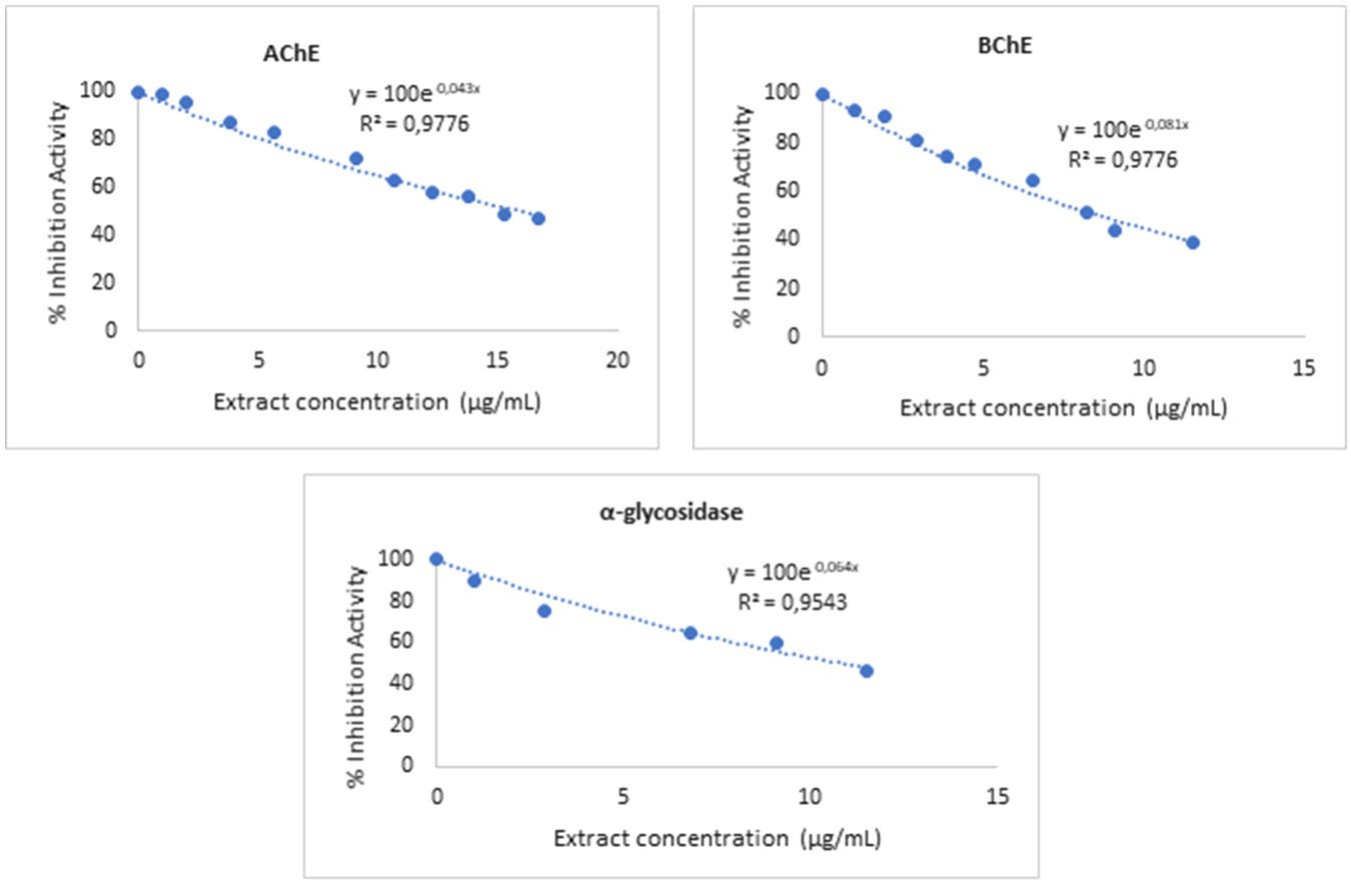
Inhibition effect of *Mangifera indica* L. ethanol extract on AChE, BChE, and α‐glucosidase.

Figure 1 illustrates the concentration‐dependent inhibitory effects of MLs ethanol extract on AChE, BChE, and α‐glucosidase enzymes. The graphs clearly demonstrate that enzyme activity decreases as the extract concentration increases. For AChE, the inhibition curve showed a good fit with the equation *y* = 100e^−0.037xy^ and an *R* ^2^ value of 0.9216, with an IC_50_ value calculated as 16.116 μg/mL. BChE inhibition exhibited a stronger correlation, described by the equation *y* = 100e^−0.081xy^ with an *R* ^2^ value of 0.9848, and an IC_50_ value of 8.56 μg/mL, indicating a more potent effect on BChE compared to AChE. Similarly, for α‐glucosidase, the inhibition curve was modeled by the equation *y* = 100e^−0.064xy^ with an *R* ^2^ value of 0.9646, and an IC_50_ value calculated as 10.828 μg/mL (Table 3 and Figure 1).

**TABLE 3:** Inhibition effect of *Mangifera indica* ethanol extract on AChE, BChE, and α‐glucosidase.

Inhibitors	AChE IC_50_ (μg/mL)	*R* ^2^	BChE IC_50_ (μg/mL)	*R* ^2^	α‐Glucosidase IC_50_ (μg/mL)	*R* ^2^
*Mangifera indica*	16.116	0.922	8.556	0.984	10.828	0.9649
Tacrine	3.19	0.989	2.54	0.987		
Acarbose					3.15	0.9961

These findings in Figure 1 and Table 3 highlight the selective and dose‐dependent effects of the extract on enzyme inhibition with reported IC_50_ values. The stronger inhibitory effect on BChE suggests a potential role in the treatment of neurodegenerative diseases, while the inhibition of α‐glucosidase supports its potential application in managing metabolic disorders such as diabetes. The high *R* ^2^ values across all graphs reinforce the accuracy and reliability of the data and analysis.

## 4 Discussion

In this study, the phenolic compound content, antioxidant capacity, and enzyme inhibition potential of MLs ethanol extract were comprehensively analyzed. The findings revealed that MLs are rich in phenolic compounds and possess health‐promoting properties. The LC–MS/MS analysis showed that the MLs extract is particularly rich in bioactive compounds such as vanillic acid, fumaric acid, gallic acid, butein, and quercetin. These compounds highlight the potential of MLs as a bioactive resource in mitigating oxidative stress and managing metabolic diseases. Antioxidant capacity tests demonstrated that the MLs extract exhibits certain antioxidant activities. Although FRAP and CUPRAC assays revealed relatively low metal activity of the MLs' extract compared to standard antioxidants such as BHA, BHT, and Trolox, it could be said that it has a moderate metal reducing capacity as a natural antioxidant agent. This is in contrast to the DPPH and ABTS assays, which confirmed the extract's radical scavenging activities. This suggests that the antioxidant activity of the MLs' extract depends not only on the concentration of phenolic compounds but also on the synergistic effects among these compounds, the matrix structure of the extract, and its bioavailability (Rasoanaivo et al. 2011).

The results align with those of Kumar et al. (2021), who identified mangiferin, quercetin, and isoquercitrin as key phenolic compounds with high antioxidant capacities in mango leaves. In Mohan et al. (2013)'s study, the IC_50_ value of the methanol extract for DPPH assay was reported as 13.37 μg/mL, whereas the DPPH radical scavenging activity of the extract in this study was recorded as 26.87% at 0.2 μg mL^−1^. Moreover, literature highlights the ethyl acetate fraction's superior antioxidant capacity, with DPPH values reaching 1226 mg Trolox equivalents/g (Kitbumrungsart et al. 2011). In contrast, the FRAP value in this study was limited to 0.118 absorbance, which could be attributed to differences in extraction methods and solvents (Kitbumrungsart et al. 2011). Beyond the antioxidant activities, the potential of mango leaves stems not only from the individual effects of phenolic compounds but also from the synergistic interactions among components. While Gu et al. (2019) emphasized the significant antioxidant contributions of mangiferin and benzophenone derivatives, this study highlights the roles of vanillic acid and quercetin in the antioxidant profile of mango leaves. Enzyme inhibition analyses revealed that the MLs extract exhibited dose‐dependent inhibitory effects on AChE, BChE, and α‐glucosidase enzymes. Particularly, the inhibition effect on AChE (IC_50_: 16.116 μg/mL) and BChE (IC_50_: 8.556 μg/mL) suggests the potential therapeutic application of MLs in the treatment of neurodegenerative diseases such as Alzheimer's. Additionally, α‐glucosidase inhibition (IC_50_: 10.83 μg/mL) indicates promising applications for managing metabolic diseases like diabetes. These findings support the value of MLs as a natural product in therapeutic applications related to neuroprotection and metabolic disorders.

Compared to existing literature, the results of this study provide novel contributions. For instance, Pan et al. (2018) highlighted the antioxidant and antidiabetic activities of benzophenone derivatives isolated from MLs, while Sferrazzo et al. (2022) focused on the antibacterial and anti‐inflammatory effects of MLs and their cellular protective roles. Das et al. (2011) emphasized the role of mangiferin as a powerful antioxidant and an effective agent against oxidative stress‐related diseases. In a study, the inhibitory effect of aqueous extract of mango leaves collected from Mumbai, India, against the α‐amylase and the α‐glucosidase enzymes as an antidiabetic was screened. MLs water extract showed an inhibitory effect against these enzymes with IC_50_ values of 132.27 ± 4.23 μg/mL and 345.79 ± 6.22 μg/mL, respectively (Kulkarni and Rathod 2018). In another study, mango leaf (Sicilian mango, collected from Lentini, Italy) extract was found to exhibit α‐glucosidase inhibitory activity with an IC_50_ value of 187.48 μg/mL (Sferrazzo et al. 2022). In this study, the ethanol extract of MLs collected from Adana province of Turkey has a higher inhibitory effect on α‐glucosidase enzyme with an IC_50_ value of 10.828 μg/mL. Based on the results of the study, the extracts are believed to have antidiabetic and antioxidant qualities since they include high concentrations of natural substances including quercetin, fumaric acid, gallic acid, butein, and vanillic acid. In this respect, the study can contribute to important studies in the literature by providing a broader perspective on the therapeutic potential of MLs.

This study also highlights that MLs offer a unique bioactive compound profile compared to mango pulp. While Palafox‐Carlos et al. (2012) investigated the roles of phenolic compounds in the oxidative metabolism of mango pulp, and Maldonado‐Celis et al. (2019) explored the nutritional properties of mango fruit, our study focuses on the therapeutic potential of mango leaves in managing neurodegenerative and metabolic diseases. The inhibitory effect on AChE and BChE further positions MLs as a natural source for treating Alzheimer's disease and similar neurodegenerative disorders.

These results underscore the extensive potential applications of MLs within the domains of health and food sciences. The detailed phenolic compound profile, coupled with the demonstrated biological activities, highlights the therapeutic value of this natural resource. Nevertheless, further validation through in vivo and clinical studies is essential to deepen our understanding of the health‐related applications and mechanistic roles of MLs. This study presents a thorough evaluation of the bioactive properties and phenolic composition of MLs, thereby making a substantial contribution to the existing body of scientific literature.

## 5 Conclusion

This study evaluated the phenolic compound content, antioxidant capacities, and enzyme inhibition effects of MLs, demonstrating their significant potential for health and industrial applications. The presence of phenolic compounds such as vanillic acid, gallic acid, and quercetin underscores the potential of these leaves as a powerful natural resource for combating oxidative stress and treating neurodegenerative diseases. The observed inhibition effects on BChE and α‐glucosidase indicate the important role of MLs in therapeutic applications related to both neuroprotection and metabolic disorders. These findings highlight the sustainable use of the bioactive properties of MLs, providing a foundation for the development of innovative products in both the food and pharmaceutical industries. The study contributes to the growing body of evidence supporting the use of natural bioactive sources in therapeutic and industrial applications. However, advanced research is required to comprehensively validate the clinical applicability of these results. Despite the promising outcomes, certain limitations must be addressed. The study's in vitro nature calls for in vivo and clinical trials to confirm the efficacy and safety of MLs extracts in real‐world applications. Additionally, factors such as bioavailability, dosage optimization, and potential toxicity need to be rigorously examined. Nevertheless, this research provides a significant contribution to the literature by highlighting the potential of MLs leaves as a natural and sustainable source of bioactive compounds. It paves the way for further exploration into their therapeutic and industrial applications, offering new opportunities for innovative product development.

## Author Contributions

**Mesut Işık:** investigation (equal), project administration (equal), writing – original draft (equal), writing – review and editing (equal). **Emrah Dikici:** investigation (equal), methodology (equal), resources (equal), validation (equal). **Sevgi Altın:** methodology (equal). **Cemalettin Alp:** methodology (equal), visualization (equal). **Kevser Kübra Kırboğa:** investigation (equal), visualization (equal), writing – original draft (equal). **Ekrem Köksal:** conceptualization (equal), supervision (equal), writing – review and editing (equal). **Şükrü Beydemir:** project administration (equal), supervision (equal).

## Conflicts of Interest

The authors declare no conflicts of interest.

## Supporting information


Appendix S1.
**Table S1.** Rectilinear regression equations and the linearity ranges of the studied standard compounds.
**Figure S1.** Chromatograms of phenolic compounds in the ethanol extract of *Mangifera indica* L.

## Data Availability

Data will be made available on request.
