# Supplementary material for: Phenolic Content, Antioxidant Capacity, and Therapeutic Potential of Mango (Mangifera indica L.) Leaves
Source: Food Sci Nutr. 2025 May 10;13(5):e70263. doi: 10.1002/fsn3.70263 (PMC12064990; doi:10.1002/fsn3.70263)
Supplement: Supplementary file 1 — Appendix S1. Table S1. Rectilinear regression equations and the linearity ranges of the studied standard compounds. Figure S1. Chromatograms of phenolic compounds in the ethanol extract of Mangifera indica L. [file FSN3-13-e70263-s001.docx]

**Table S1.** Rectilinear regression equations and the linearity ranges of the studied standard compounds

| Standard compounds | ESI IYON MOD | ^a^MRM | ^b^RSD % | ^c^RT | ^d^R^2^ | Regression |
| --- | --- | --- | --- | --- | --- | --- |
| Quercetin | neg | 301,1>151 | 0.0136 | 3,890 | 0,999 | Y=(13,7831)X+(-146,951) |
| Acetohydroxamic acid | pos | 76,10>43,10 | 0.0082 | 0,398 | 0,999 | Y = (150,982)X + (23,1833) |
| Catechin hydrate | Neg | 291,10>139,00 | 0.0236 | 2,722 | 0,999 | Y = (79,2933)X + (-2406,22) |
| Vanillic acid | pos | 168,80>93,00 | 0.0062 | 2,885 | 0,998 | Y = (48,0522)X + (-876,904) |
| Resveratrol | pos | 229,10>135,00 | 0.0131 | 4,314 | 0,998 | Y = (46,4361)X + (-1314,61) |
| Fumaric acid | neg | 115,20>71,00 | 0.0047 | 0,507 | 0,999 | Y = (20,2986)X + (-762,592) |
| Gallic acid | neg | 169,20>125,00 | 0.0136 | 1.442 | 0,999 | Y = (65,3835)X + (-2699,84) |
| Caffeic acid | neg | 179,20>135,00 | 0.0137 | 2,778 | 0,996 | Y = (124,785)X + (-487,132) |
| Phloridzin dyhrate | neg | 435,00>273,10 | 0.0564 | 3,462 | 0,999 | Y = (33,4069)X + (-1396,90) |
| Oleuropein | neg | 539,10>377,20 | 0.0694 | 3.567 | 0,999 | Y = (25,9240)X + (-558,916) |
| Hydroxycinnamic acid | neg | 163,20>119,00 | 0.0856 | 3,900 | 0,995 | Y = (13,1516)X + (717,421) |
| Ellagic acid | neg | 300,90>145,10 | 0.0079 | 5.017 | 1,000 | Y = (5,25903)X + (-1167,31) |
| Myricetin | neg | 317,10>150,90 | 0.0129 | 3.556 | 0,999 | Y = (37,0934)X + (2684,23) |
| Protocatechuic acid | neg | 181,20>108,00 | 0.0145 | 3,853 | 0,994 | Y = (526,954)X + (23026,1) |
| Bütein | neg | 271,10>135,00 | 0.0205 | 3,879 | 0,999 | Y = (49,3543)X + (367,917) |
| Naringenin | neg | 271,10>150,90 | 0.0057 | 4,124 | 0,996 | Y = (317,241)X + (33733,3) |
| Luteolin | neg | 285,20>132,90 | 0.0144 | 4,115 | 0,998 | Y = (34,6668)X + (3721,79) |
| Kaempferol | neg | 285,10>116,90 | 0.0351 | 4.594 | 0,999 | Y = (2,63905)X + (-206,494) |
| Alizarin | neg | 239,20>210,90 | 0.0856 | 3.528 | 0,998 | Y = (3,97487)X + (1614,23) |
| Hydroxybenzoic acid | neg | 137,20>93,00 | 0.0079 | 4.212 | 0,999 | Y = (735,804)X + (-498,102) |

^a^MRM: Multiple Reaction Monitoring. ^b^RSD: Relative standard deviation. ^c^RT: Retention time.^d^R^2^: Determination coefficient.

**MS Chromatogram**


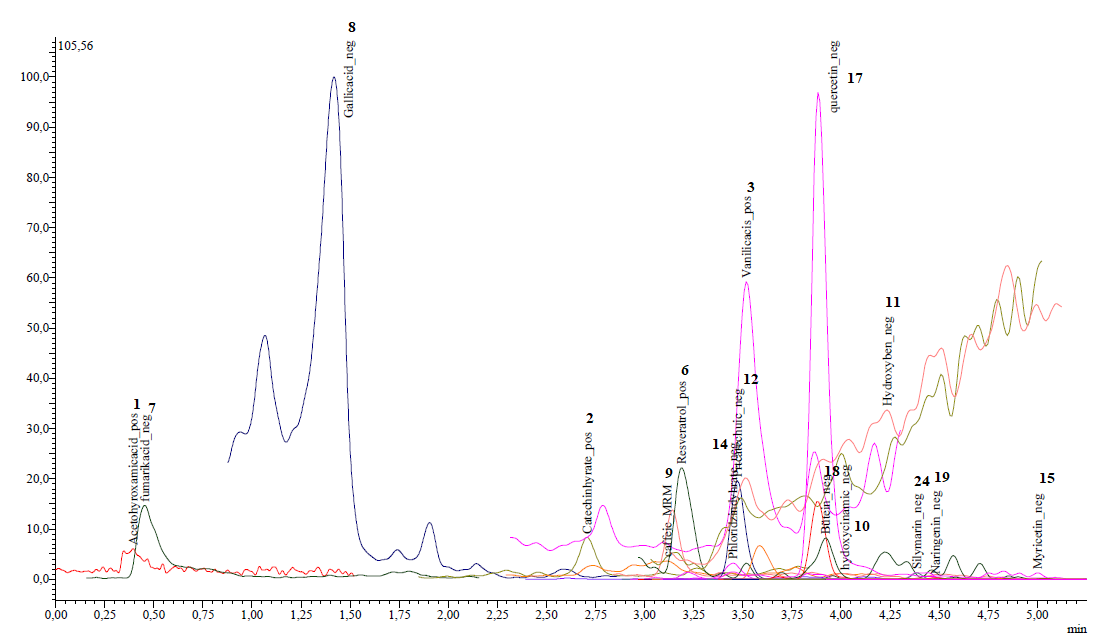


**Figure S1.** Chromatograms of phenolic compounds in the ethanol extract of *Mangifera indica* L.

| **Number** | **Name** | **Number** | **Name** |
| --- | --- | --- | --- |
| 1 | Acetohydroxamic acid _poz | 13 | Oleuropein_neg |
| 2 | Catechin hydrate_pos | 14 | Phloridzin dyhrate_neg |
| 3 | Vanilic acid_poz | 15 | Myricetin_neg |
| 4 | Syringic acid_pos | 16 | Ellagic acid_neg |
| 5 | Thymoquinone_poz | 17 | Quercetin_neg |
| 6 | Resveratrol_pos | 18 | Bütein_neg |
| 7 | Fumaric acid _neg | 19 | Naringenin_neg |
| 8 | Gallic acid_neg | 20 | Luteolin_neg |
| 9 | Caffeic acid_MRM | 21 | Kaempferol_neg |
| 10 | Hydroxycinnamic acid_neg* | 22 | Alizarin_neg |
| 11 | Hydroxybenzoic acid_neg | 23 | Curmin_neg |
| 12 | Protocatechuic acid_neg | 24 | Silymarin_neg |
